# What is normal age-related thigh muscle composition among 45- to 84-year-old adults from the UK Biobank study

**DOI:** 10.1007/s11357-024-01304-y

**Published:** 2024-08-12

**Authors:** David B. Anderson, Aaron J. Beach, Lingxiao Chen, Henry J. Feng, Marnee J. McKay, Zachary A. Smith, Kenneth A. Weber, Evert Onno Wesselink, James M. Elliott

**Affiliations:** 1 School of Health Sciences, Faculty of Medicine and Health, The University of Sydney, Sydney, Australia; 2 Department of Health Sciences, Faculty of Medicine, Health and Human Sciences, Macquarie University, Sydney, Australia; 3 Department of Orthopaedics, Shandong University Centre for Orthopaedics, Qilu Hospital, Cheeloo College of Medicine, Shandong University, Jinan, Shandong 250012 People’s Republic of China; 4 Department of Biostatistics, School of Public Health, Cheeloo College of Medicine, Shandong University, Jinan, Shandong 250012 People’s Republic of China; 5 Division of Psychology and Mental Health, Faculty of Biology, Medicine and Health, University of Manchester, Manchester, M13 9PL UK; 6 University of Sydney, Faculty of Medicine and Health and the Northern Sydney Local Health District, The Kolling Institute, Sydney, Australia; 7 University of Oklahoma Health Sciences Center, Oklahoma City, OK 73116 USA; 8 Division of Pain Medicine, Department of Anaesthesiology, Perioperative and Pain Medicine, Stanford University School of Medicine, Palo Alto, CA USA; 9 Faculty of Behavioural and Movement Sciences, Amsterdam Movement Sciences, Vrije Universiteit Amsterdam, Amsterdam, The Netherlands

**Keywords:** Pain, Neuromuscular disease, Skeletal muscle mass, Muscle composition, Fat-free muscle

## Abstract

A loss of skeletal muscle mass and an increase in intramuscular fat are known to occur as we enter middle and older age, but the expected changes or normative values have remained unknown. The primary reason for this is that imaging studies are difficult and expensive to conduct, and consequently, the sample sizes have remained small. The development of the UK Biobank which provides access to a large magnetic resonance imaging (MRI) data set of more than 50,000 participants provides an opportunity to finally address this question of normative values for each age group. The study’s primary aim was to determine the age-related changes in thigh muscle composition (e.g., thigh fat-free muscle volume and intramuscular fat) between the ages of 45 and 84 years. The second aim was to analyse associations between thigh fat-free muscle volume and intramuscular fat with lifestyle behaviours (smoking, alcohol consumption, and physical activity), leg pain, and bone mineral density. Fifty thousand three hundred thirty-two participants were included in the study. Total fat-free thigh muscle declined between the ages of 45 and 84 years, while intramuscular fat of the thigh continued to increase. The changes were stable between these age groups. The mean volume of fat-free muscle ranged from 11.16 (SD: 1.40) to 13.26 L (SD: 1.85) in adult males and 7.60 (SD: 0.97) to 8.80 L (SD 1.29) in females between the ages of 45 and 84 years. For intramuscular fat, the change among women was from 6.94% (SD: 1.59) in the 45 to 54 years age bracket to 8.83% (SD: 1.92) in the 75 to 84 age bracket, while for men, it was 5.83% (SD: 1.30) in the 45 to 54 age bracket to 7.85% (SD 1.89) in the 75 to 84 age bracket. The total fat-free muscle volume and intramuscular fat percentage provided can be used for the purpose of reference standards or normative values for adults in the age groups provided. Fat-free muscle and intramuscular fat were found to be associated with a range of health, activity, and leg pain outcomes, and these should be investigated in a follow-up longitudinal imaging study.

**Supplementary Information:**

The online version contains supplementary material available at 10.1007/s11357-024-01304-y.

## Introduction

This progressive loss of muscle mass is ubiquitous across all mammalian species and common in adults aged 65 years and older [1, 2]. Recent analyses suggest that muscle loss is a natural phenomenon of ageing [3]; while others suggest that muscle loss is a disease due to its association with health-related outcomes [4, 5]. Intramuscular fat has also been considered an important marker of health, however, like muscle mass, its impact on health-related outcomes remains unclear [6, 7]. Muscle mass and intramuscular fat have been found previously to be associated with a range of pain and health conditions [8–12]. Unfortunately, these studies are limited by smaller sample sizes with imprecise estimates and low statistical power.

To increase our understanding of changes in muscle-mass (specifically fat-free muscle) and intramuscular fat as we age, studies with large sample sizes are needed. The UK Biobank offers a unique opportunity to overcome the limitations of the previous studies of fat-free muscle and intramuscular fat by providing access to the imaging of over 50,000 participants [13]. This dataset far exceeds the sample size of previous imaging studies and creates the opportunity to contribute to the development of a normative reference standard for muscle composition that could allow adults to compare their muscle composition to others of the same age bracket.

One major consideration for researchers using the UK Biobank is which muscle group to select when identifying muscle composition. For this study, the thigh was selected for identifying fat-free muscle volume and intramuscular fat as the thigh is able to provide reliable estimates with the available imaging data in the UK Biobank, and its precedent as a representative region of muscle composition in a person [10, 11].

Given the focus of previous studies [8–12] on the association of fat-free muscle and intramuscular fat on health outcomes, a secondary aim was to analyse any associations between a range of behavioural outcomes (smoking use and alcohol consumption), self-reported physical function (i.e., walking, duration of strenuous activity), the clinical outcomes of bone mineral density, and leg pain with muscle composition. The primary aim of the study was to present the mean total fat-free muscle volume and intramuscular fat between the ages of 45 and 84 years to generate sex-related normative values and reference standards for the community.

## Methods

### Study participants

This study analysed the currently available imaging data of 50,332 subjects from the UK Biobank, acquired between 2014 and 2022, with patient meta-data obtained through application number 86983. The UK Biobank is a large prospective study of over 500,000 volunteers between 40 and 84 years across the UK, collecting data on genetics, biochemistry, health and lifestyle, and medical records [13]. A subset of individuals also volunteered for the whole-body imaging study, with a planned 100,000 participants. The study received ethical approval from the North West Multi-Centre Research Ethics Committee (REC reference: 16/NW/0274) and was conducted in accord with the principles of the Declaration of Helsinki. All participants provided written informed consent for data collection, analysis, and record linkage. The study followed the STROBE reporting guidelines.

Participants from the UK Biobank meeting the inclusion and none of the exclusion criteria were included.

Inclusion criteria was as follows:

- An adult (female or male) who had enrolled in and completed data collection for the UK Biobank.
- Had imaging data available (the initial imaging assessment (Instance 2) of the UK Biobank.

Exclusion criteria was as follows:

- Did not have data available on the primary and secondary outcomes at the initial imaging assessment follow-up.

### Imaging protocol

The UK Biobank MRI protocol examined in this study is listed as UK Biobank field 20,201 and detailed in supplementary documentation. All participants were scanned in a Siemens MAGNETOM Aera 1.5-T MRI scanner (Siemens Healthineers, Erlangen, Germany) at one of six centres. Volumetric, co-aligned images of water and fat signal were acquired with a 6-min dual-echo Dixon Vibe protocol with TR = 6.69, TE 1 and 2 = 2.39/4.77 ms, and flip angle 10° [14, 15]. Each whole-body data set consists of 6 scan positions covering the neck, thorax, abdomen, pelvis, and thighs. The image resolution varied between stations, with a typical grid of 224 × 174 × 44 and voxel size of 2.232 × 2.232 × 4.5 mm. Full-body DEXA (General Electric Lunar iDXA, Madison, WI) is also completed with staff trained to standardise assessment and using previously validated methods [16].

### Image analysis

Derived measures of body composition were developed by AMRA™ Profiler (Advanced MR Analytics, Linköping, Sweden), based on volumetric multi-atlas segmentations [14, 17]. These included abdominal subcutaneous adipose tissue (ASAT), visceral adipose tissue (VAT), and posterior and anterior thigh muscle mass (right and left) and measured in litres as described in Borga et al. [17]. The total abdominal adipose tissue (TAAT) is separated into intra-abdominal (IAAT) and abdominal subcutaneous (ASAT) adipose tissue. The volume of the thigh muscles is measured in litres as described in Karlsson et al. [18]. The muscle volume is defined as the lean muscle volume, being the volume enclosing the muscle subtracted by the volume of adipose tissue within the same volume. The anterior thigh muscle group comprised of the quadriceps femoris and sartorius. The posterior thigh muscle is comprised of the hamstring, gluteus, iliacus, and adductor.

### Exposures

Fat-free muscle volume was defined as the volume of all voxels with fat fraction < 50% in the thighs. Intramuscular fat was defined as the mean fat fraction in the ‘viable muscle tissue’ of the right and left anterior thighs. These definitions have been used in previous UK Biobank publications [19].

### Outcomes

The primary outcomes were (a) the total thigh fat-free muscle volume and (b) intramuscular fat of participants characterised by age (45–54, 55–64, 65–74, and 75–84) from fat–water Dixon MRI. Secondary outcomes included associations for total thigh fat-free muscle volume or intramuscular fat with bone mineral density (total, legs), smoking status, alcohol consumption, leg pain, and physical activity. Leg pain included eight variables: leg pain on walking, leg pain when standing still or sitting, leg pain in calf/calves, leg pain when walking uphill or hurrying, leg pain when walking normally, leg pain when walking ever disappears while walking, leg pain on walking: action taken, and leg pain on walking: effect of standing still. Physical activity included eight duration-related variables (duration of heavy Do-It-Yourself (DIY), duration of light DIY, duration of moderate activity, duration of other exercises, duration of strenuous sports, duration of vigorous activity, duration of walks, and duration walking for pleasure) and six frequency-related variables (frequency of heavy DIY in last 4 weeks, frequency of light DIY in last 4 weeks, frequency of other exercises in last 4 weeks, frequency of stair climbing in last 4 weeks, frequency of strenuous sports in last 4 weeks, and frequency of walking for pleasure in last 4 weeks) (see Supplementary Table 1A for full list).

### Statistical analysis

For the primary outcome, the baseline characteristics were tabulated with means and standard deviations presented for continuous variables. For the categorical variables, the frequency of responses, the percentage of the cohort, and the percentage of total responses are presented.

For the secondary outcome, we examined the association between muscle composition (total thigh fat-free muscle volume and intramuscular fat) and all above mentioned outcomes through a stepped modelling framework: model 1, unadjusted analyses; model 2, analyses adjusted for age and sex (see Tables 3 and 4). Logistic regression was used when the outcome was binary (e.g., smoking status) and the corresponding estimates were reported as odds ratio (OR) and 95% confidence interval (CI); linear regression was used when the outcome was continuous and the corresponding estimates were reported as mean difference (MD) and 95% CI; ordinal logistic regression was used when the outcome was ordinal (e.g., frequency of walking for pleasure in last 4 weeks) and the corresponding estimates were reported as proportional OR and 95% CI. All statistical analyses were performed in R, version 4.2.2 (R Group for Statistical Computing).

### Patient and public involvement

No patients or public involvement was present in our study.

## Results

The study included 50,332 participants from the UK Biobank study who consented and underwent imaging that included the thigh. Baseline characteristics of the participants are outlined in Table 1, with the full table provided in supplementary material (see Table 1a).

**Table 1:** Baseline characteristics

Cohort characteristics	*N* = 50,332	% total responses
Age (mean ± SD)	64.72 (7.7)	
Gender		
Male	24,266	48.21%
Female	26,066	51.80%
Ethnicity*		
British	45,773	90.97%
Any other white background	1,524	3.03%
Irish	1,333	2.65%
Indian	368	0.73%
Caribbean	181	0.36%
Chinese	146	0.29%
African	139	0.28%
Prefer not to answer	115	0.23%
Ethnic group not listed	753	0.53%
BMI (mean ± SD)	26.53 (4.4)	
***Outcomes***		
Smoking status		
Current	1,662	3.3%
Previous	17,005	34.0%
Never	31,132	62.3%
Prefer not to answer	186	0.4%
Alcohol consumption		
Daily or almost daily	8,545	17.1%
Three or four times per week	13,961	27.9%
Once or twice a week	13,162	26.3%
One to three times per month	5,677	11.4%
Special occasions	5,196	10.4%
Never	3,425	6.9%
Prefer not to answer	19	< 0.1%
Alcohol units (per day) (SD)	1.08 (1.21)	
Leg pain when standing still or sitting		
Yes	5,246	52.5%
No	4,398	44.3%
Do not know	315	3.1%
Prefer not to answer	4	< 0.1%
Leg pain when walking		
Yes	9,963	19.9%
No	39,643	79.3%
Do not know	304	0.6%
Prefer not to answer	75	0.2%
Total thigh fat-free muscle volume (mean ± SD)	10.15 (2.52)	
Total adipose tissue volume (mean ± SD)	20.86 (7.03)	
Total lean tissue volume (mean ± SD)	24.10 (4.79)	
Intramuscular fat (mean of anterior left and right only) (mean ± SD)	7.32 (1.87)	
Weight-to-muscle ratio (mean ± SD)	7.62 (1.33)	
Total BMD (mean ± SD)	1.20 (0.15)	
Total BMD (*T*-score) (mean ± SD)	0.59 (1.24)	
Legs BMD (mean ± SD)	1.23 (0.18)	
Pelvis BMD (mean ± SD)	1.00 (0.15)	

*BMD* bone mineral density, *SD*, standard deviation

^*^Full list provided in supplementary materials as Table 1A

**Fig. 1:**
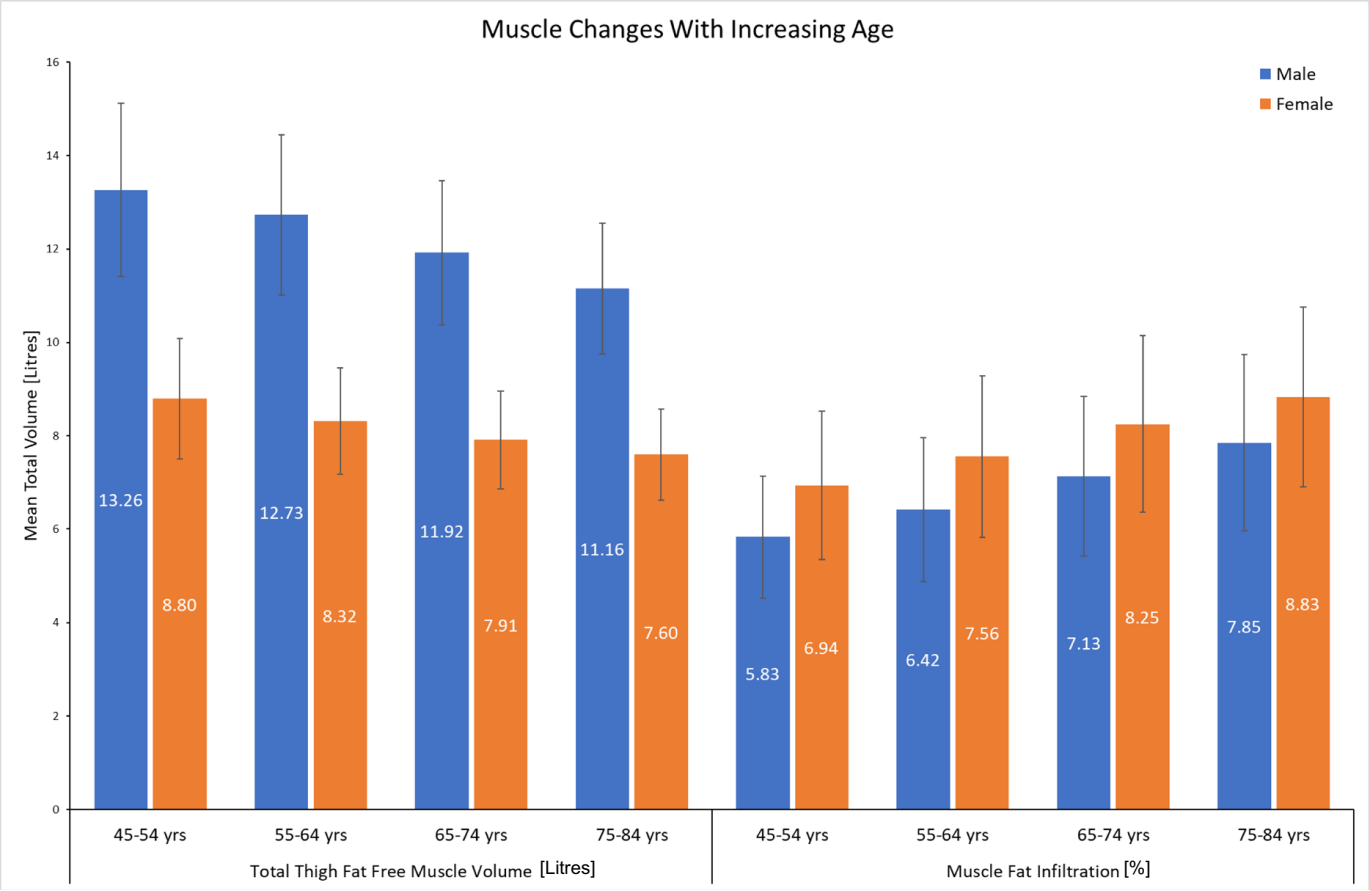
Muscle composition change with increasing age

In both females and males, the peak volume of thigh fat-free muscle was in the 45 to 54 age group (8.80 L in females and 13.26 L in males) and continued a linear decline, with the lowest volumes recorded in the 75 to 84 age group (7.60 L in females and 11.16 L in males). Increasing age was related to increasing intramuscular fat, with the lowest intramuscular fat recorded in the 45 to 54 age group for both females and males (6.94% in females and 5.83% in males) and highest recorded in the 75 to 84 age group (8.83% in females and 7.85% in males) (Table 2, Fig. 1).

**Table 2:** Characteristics of thigh muscle composition categorised by age and sex

Sex	Age group	*N*	BMI	Total thigh fat-free muscle volume (litres)	Intramuscular fat (%)
Female					
	45–54	3222	26.23	8.80 (± 1.29)	6.94 (± 1.59)
	55–64	10,053	26.32	8.32 (± 1.14)	7.56 (± 1.73)
	65–74	10,494	25.92	7.91 (± 1.04)	8.25 (± 1.88)
	75–84	2297	25.76	7.60 (± 0.97)	8.83 (± 1.92)
Male					
	45–54	2536	27.18	13.26 (± 1.85)	5.83 (± 1.30)
	55–64	8020	27.23	12.73 (± 1.71)	6.42 (± 1.54)
	65–74	10,552	26.92	11.92 (± 1.55)	7.13 (± 1.71)
	75–84	3158	26.45	11.16 (± 1.40)	7.85 (± 1.89)

Results presented as the mean ± standard deviation

### Association between fat-free muscle volume and outcomes

#### Smoking and physical activity

Table 3 identifies the association between fat-free muscle volume and selected outcomes. Participants who smoked were less likely to have higher fat-free muscle volume (OR 0.92, 95% CI 0.88 to 0.96). Participants who reported longer durations of vigorous activity (MD 1.40, 95% CI 1.02 to 1.78), spent longer time walking for pleasure (OR 1.05, 95% CI 1.04 to 1.07), spent longer time doing other exercises (OR 1.09, 95% CI 1.07 to 1.12), spent longer time doing light (OR 1.02, 95% CI 1.00 to 1.04) and heavy DIY activities (OR 1.05, 95% CI 1.03 to 1.07), more frequently doing other exercises (OR 1.10, 95% CI 1.08 to 1.12), more frequently climbing a flight of stairs (OR 1.01, 95% CI 1.00 to 1.02), and more frequently doing strenuous sports (OR 1.11, 95% CI 1.07 to 1.15) were more likely to have higher fat-free muscle volume.

**Table 3:** Association between total fat-free muscle volume and outcomes, adjusted and unadjusted for age and sex

Outcome	Adjusted model Odds ratio (95%CI)	Unadjusted model Odds ratio (95%CI)
*Modifiable lifestyle behaviours*		
Smoking status	0.92 (0.88 to 0.96)	1.06 (1.04 to 1.09)
Alcohol use	1.06 (1.04 to 1.08)	1.13 (1.12 to 1.14)
Physical activity		
Duration of walks	MD − 0.88 (− 1.40 to − 0.35)	MD − 0.53 (− 0.81 to − 0.24)
Duration walking for pleasure	1.05 (1.04 to 1.07)	1.01 (1.00 to 1.02)
Duration of moderate activity	MD 0.13 (− 0.41 to 0.67)	MD − 0.52 (− 0.81 to -0.22)
Duration of vigorous activity	MD 1.40 (1.02 to 1.78)	MD 1.04 (0.84 to 1.25)
Duration of other exercises	1.09 (1.07 to 1.12)	1.04 (1.03 to 1.05)
Duration of strenuous sports	1.01 (0.97 to 1.05)	1.04 (1.02 to 1.06)
Duration of light DIY	1.02 (1.00 to 1.04)	1.05 (1.04 to 1.07)
Duration of heavy DIY	1.05 (1.03 to 1.07)	1.08 (1.06 to 1.09)
Frequency of light DIY in last 4 weeks	1.00 (0.98 to 1.02)	0.99 (0.98 to 1.00)
Frequency of heavy DIY in last 4 weeks	1.01 (0.99 to 1.03)	1.03 (1.02 to 1.04)
Frequency of other exercises in last 4 weeks	1.10 (1.08 to 1.12)	1.06 (1.05 to 1.07)
Frequency of stair climbing in last 4 weeks	1.01 (1.00 to 1.02)	1.00 (0.99 to 1.01)
Frequency of strenuous sports in last 4 weeks	1.11 (1.07 to 1.15)	1.04 (1.02 to 1.07)
Frequency of walking for pleasure in last 4 weeks	0.97 (0.96 to 0.99)	0.98 (0.97 to 0.99)
*Bone mineral density*	MD 0.032 (0.031 to 0.033)	MD 0.041 (0.041 to 0.041)
*Leg pain*		
Leg pain on walking	1.05 (1.03 to 1.07)	1.00 (0.99 to 1.01)
Leg pain on walking: action taken	0.92 (0.87 to 0.98)	0.99 (0.96 to 1.03)
Leg pain on walking: effect of standing still	1.00 (0.96 to 1.04)	0.98 (0.95 to 1.00)
Leg pain when walking ever disappears	1.03 (0.99 to 1.09)	1.02 (0.99 to 1.05)
Leg pain when walking normally	0.98 (0.95 to 1.02)	0.98 (0.96 to 1.00)
Leg pain when walking uphill or hurrying	0.92 (0.88 to 0.95)	0.93 (0.91 to 0.95)
Leg pain in calf/calves	0.92 (0.88 to 0.96)	1.00 (0.98 to 1.02)
Leg pain when standing still or sitting	1.00 (0.96 to 1.03)	0.98 (0.96 to 1.00)

The adjusted model included results adjusted for age and sex and was the primary model used

*MD* mean difference

#### Leg pain

People that reported stopping or slowing down when they get pain during walking (OR 0.92, 95% CI 0.87 to 0.98), getting pain when they walk uphill or hurry (OR 0.92, 95% CI 0.88 to 0.95), and getting pain in their calves (OR 0.92, 95% CI 0.88 to 0.96) were less likely to have higher fat-free muscle volume. People reporting pain in either leg on walking were associated with lower fat-free muscle volume (OR 1.05, 95% CI 1.03 to 1.07) (see Table 3A in supplementary for full list).

### Association between intramuscular fat and outcomes

#### Smoking and physical activity

Table 4 identified associations between intramuscular fat and selected outcomes. Participants who smoked were found to be more likely to have higher intramuscular fat (OR 1.16, 95% CI 1.13 to 1.20). People who reported shorter durations of walks (MD − 0.95, 95% CI − 1.38 to − 0.52), moderate activity (MD − 1.19, 95% CI − 1.63 to − 0.74), and vigorous activity (MD − 1.53, 95% CI − 1.86 to − 1.20) were associated with lower odds of increased intramuscular fat. Spending less time: walking for pleasure (OR 0.87, 95% CI 0.86 to 0.88), doing other exercises (OR 0.92, 95% CI 0.91 to 0.94), doing light (OR 0.98, 95% CI 0.97 to 0.99) and heavy (OR 0.96, 95% CI 0.94 to 0.98) DIY, climbing a flight of stairs (OR 0.86, 95% CI 0.85 to 0.87), and less frequently doing strenuous sports (OR 0.85, 95% CI 0.82 to 0.89) were also associated with lower odds of higher intramuscular fat.

**Table 4:** Association between intramuscular fat and outcomes, adjusted and unadjusted for age and sex

Outcomes	Adjusted model Odds ratio (95%CI)	Unadjusted model Odds ratio (95%CI)
*Modifiable lifestyle behaviours*		
Smoking status	1.16 (1.13 to 1.20)	1.05 (1.02 to 1.08)
Alcohol use	0.96 (0.95 to 0.97)	0.93 (0.92 to 0.94)
Physical activity		
Duration of walks	MD − 0.95 (− 1.38 to − 0.52)	MD − 0.98 (− 1.37 to − 0.58)
Duration walking for pleasure	0.87 (0.86 to 0.88)	0.91 (0.89 to 0.92)
Duration of moderate activity	MD − 1.19 (− 1.63 to − 0.74)	MD0.057 (− 0.35 to 0.47)
Duration of vigorous activity	MD − 1.53 (− 1.86 to − 1.20)	MD − 1.75 (− 2.05 to − 1.45)
Duration of other exercises	0.92 (0.91 to 0.94)	0.94 (0.92 to 0.95)
Duration of strenuous sports	1.03 (0.99 to 1.07)	1.05 (1.01 to 1.09)
Duration of light DIY	0.98 (0.97 to 0.99)	0.96 (0.94 to 0.97)
Duration of heavy DIY	0.96 (0.94 to 0.98)	0.92 (0.91 to 0.94)
Frequency of light DIY in last 4 weeks	0.98 (0.96 to 0.99)	1.01 (1.00 to 1.03)
Frequency of heavy DIY in last 4 weeks	0.96 (0.94 to 0.98)	0.98 (0.96 to 0.99)
Frequency of other exercises in last 4 weeks	0.87 (0.86 to 0.88)	0.88 (0.86 to 0.89)
Frequency of stair climbing in last 4 weeks	0.86 (0.85 to 0.87)	0.87 (0.87 to 0.88)
Frequency of strenuous sports in last 4 weeks	0.85 (0.82 to 0.89)	0.86 (0.83 to 0.89)
Frequency of walking for pleasure in last 4 weeks	0.99 (0.97 to 1.00)	1.00 (0.99 to 1.02)
*Bone mineral densit*y	MD 0.0064 (0.0056 to 0.0072)	MD − 0.013 (− 0.014 to − 0.012)
*Leg pain*		
Leg pain on walking	1.28 (1.26 to 1.30)	1.26 (1.24 to 1.28)
Leg pain on walking: action taken	1.22 (1.18 to 1.27)	1.21 (1.17 to 1.25)
Leg pain on walking: effect of standing still	1.07 (1.04 to 1.10)	1.05 (1.02 to 1.08)
Leg pain when walking ever disappears	0.85 (0.82 to 0.88)	0.87 (0.84 to 0.90)
Leg pain when walking normally	1.11 (1.08 to 1.14)	1.10 (1.07 to 1.12)
Leg pain when walking uphill or hurrying	1.15 (1.12 to 1.18)	1.14 (1.11 to 1.17)
Leg pain in calf/calves	1.07 (1.04 to 1.10)	1.04 (1.02 to 1.07)
Leg pain when standing still or sitting	1.06 (1.03 to 1.10)	1.05 (1.02 to 1.07)

#### Leg pain

Participants who reported pain in either leg on walking (OR 1.28, 95% CI 1.26 to 1.30), stopping or slowing down when getting pain during walking (OR 1.22, 95% CI 1.18 to 1.27), experiencing pain that usually continues for more than 10 min while walking if they stand still (OR 1.07, 95% CI 1.04 to 1.10), getting pain when they walk at an ordinary pace on the level (OR 1.11, 95% CI 1.08 to 1.14), getting pain when they walk uphill or hurry (OR 1.15, 95% CI 1.12 to 1.18), getting pain in their calves (OR 1.07, 95% CI 1.04 to 1.10), and getting pain when they are standing still or sitting (OR 1.06, 95% CI 1.03 to 1.10) were associated with higher intramuscular fat. People reporting the disappearance of pain when they walk were less likely to have higher intramuscular fat (OR 0.85, 95% CI 0.82 to 0.88) (see Table 4A in supplementary for full list).

## Discussion

In this large database study (*n* = 50,332), we found that total fat-free thigh muscle declined between the ages of 45 and 84 years, while intramuscular fat of the thigh continued to increase. The changes were stable and linear between these age groups. The mean volume of fat-free muscle ranged from 11.2 to 13.3 L in adult males and 7.6 to 8.8 L in females between the ages of 45 and 84 years. For intramuscular fat, the change among women was from 6.94% in the 45 to 54 years age bracket to 8.83% in the 75 to 84 age bracket, while for men it was 5.83% in the 45 to 54 age bracket to 7.85% in the 75 to 84 age bracket. The results demonstrate that on average men experience a greater loss of fat-free muscle compared with women between the ages of 45 to 54 and 75 to 84 (15.83% reduction for men and 13.63% reduction for women). Men also experience a greater increase in intramuscular fat compared with women between the ages of 45 to 54 and 75 to 84. Our results are supported by previous studies [20], including those that have used older versions of UK Biobank with smaller samples (i.e., 14,148 participants compared with our study of 50,332 participants) [20].

A key advantage of our study is the ability to generate very precise results due to the large sample size. The results can therefore be considered as reference value for individuals comparing their fat-free muscle and intramuscular fat values with others in their age bracket. Reference values for muscle composition serve the benefit of allowing the community to identify what changes an individual may expect as they age and to avoid being classified as having ‘abnormal changes’. Reference values are commonly in use with blood and urine tests among others, but have not yet been established for muscle composition.

Our secondary aim to analyse associations between muscle composition and selected outcomes found significant but often small associations. Interestingly, leg pain outcomes had inconsistent associations with total fat-free muscle volume, but were strongly associated with intramuscular fat, warranting further investigation. The studies should include longitudinal follow-up and should consider using available automated segmentation algorithms, which can provide time-saving measures without sacrificing measurement accuracy. Given the burden of leg pain among adults 45 years and older is more than 43% [21], targeted treatments to reduce its prevalence remain a priority﻿ [22, 23], and increased understanding of any causal relationship is worthy of investigation. Previous associations have already been reported between intramuscular fat with other conditions, such as fibromyalgia [8], lumbar spinal stenosis in those with achondroplasia [24], cervical myelopathy [9], whiplash [25], neck pain [26], and heart failure [10].

Almost all physical activity outcomes were associated with small but significant increases in total fat-free muscle volume and decreased intramuscular fat. Both increased duration of moderate and vigorous activity were associated with increased total fat-free muscle volume and decreased intramuscular fat, with the change being larger in vigorous activity. These findings are consistent with existing literature which promotes physical activity for increasing fat-free muscle and reducing intramuscular fat [27]. The impact of self-reported versus objective capture of physical activity remains unknown, but future studies would benefit from including both measures. Limitations are present in this review. The population within the UK Biobank is not ethnically diverse, which limits the generalisability of the results beyond citizens of the UK [28]. The composition of the thigh muscle relied upon the resolution of the imaging. Given the volume of imaging stored in the UK Biobank, the resolution of the imaging could be lower than in other smaller studies. The UK Biobank undertook a series of initiatives to ensure the quality of the imaging, however, such as extensive training of radiology staff, imaging quality checks, and a large data storage centre [15]. For the secondary aims, outcomes such as physical activity were self-reported, which, although common in studies, may not be representative of true function for that individual [29, 30]. The study also looked at associations, which can be impacted by confounders, such as age, sex at birth, and other life-expected comorbidities, including, but not limited to, diabetes, cardiovascular disease, neurodegenerative disorders, mental health conditions, or early life adversity. We aimed to address this limitation by including an additional analysis adjusted for age and sex, but this falls short of attempting to adequately capture the complexities of the human experience [31, 32].

## Conclusions

This study found between 45 and 84 years of age fat-free muscle continued to decline, while the magnitude of intramuscular fat increased. The mean fat-free muscle volume and intramuscular fat percentage identified in this large study support an initial reference standard for normative values. Reference standards or normative value standards can provide a comparative measure controlled for age and sex and other potentially confounding factors. Most physical activity and leg pain outcomes were associated with both fat-free muscle and intramuscular fat, and these should be further investigated in a longitudinal study, potentially with the large UK Biobank data.

## Supplementary Information

Below is the link to the electronic supplementary material.

Supplementary file1 (DOCX 57 KB)

## Data Availability

This research has been conducted using the UK Biobank Resource under Project ID: 86983. Requests to access the data should be made via application directly to the UK Biobank, https://www.ukbiobank.ac.uk.
