# Supplementary material for: What is normal age-related thigh muscle composition among 45- to 84-year-old adults from the UK Biobank study
Source: GeroScience. 2024 Aug 12;47(1):1175–85. doi: 10.1007/s11357-024-01304-y (PMC11872857; doi:10.1007/s11357-024-01304-y)
Supplement: Supplementary file 1 — Supplementary file1 (DOCX 57 KB) [file 11357_2024_1304_MOESM1_ESM.docx]

**Supplementary Files**

**Table 1A**

| **Outcomes** | **N=50,332** | **% cohort** | **% total responses** |
| --- | --- | --- | --- |
| Age | 64.72 (SD 7.7) |  |  |
| 45-49 | 829 |  | 1.65% |
| 50-54 | 4929 |  | 9.79% |
| 55-59 | 8071 |  | 16.04% |
| 60-64 | 10002 |  | 19.87% |
| 65-69 | 11286 |  | 22.42% |
| 70-74 | 9760 |  | 19.39% |
| 75-79 | 4750 |  | 9.44% |
| 80-84 | 705 |  | 1.4% |
| Gender |  |  |  |
| Male | 24266 | 48.21% | 48.21% |
| Female | 26066 | 51.80% | 51.80% |
| Ethnicity |  |  |  |
| British | 45773 | 90.94% | 90.97% |
| Any other white background | 1524 | 3.03% | 3.03% |
| Irish | 1333 | 2.65% | 2.65% |
| Indian | 368 | 0.73% | 0.73% |
| Other ethnic group | 268 | 0.53% | 0.53% |
| Caribbean | 181 | 0.36% | 0.36% |
| Chinese | 146 | 0.29% | 0.29% |
| African | 139 | 0.28% | 0.28% |
| Prefer not to answer | 115 | 0.23% | 0.23% |
| Any other Asian background | 94 | 0.19% | 0.197% |
| Any other mixed background | 79 | 0.16% | 0.165% |
| Pakistani | 78 | 0.15% | 0.16% |
| White and Asian | 72 | 0.14% | 0.14% |
| White and Black Caribbean | 57 | 0.11% | 0.11% |
| White | 30 | 0.06% | 0.065% |
| White and Black African | 28 | 0.06% | 0.06% |
| Bangladeshi | 9 | 0.02% | 0.02% |
| Do not know | 10 | 0.02% | 0.02% |
| Mixed | 5 | 0.01% | 0.01% |
| Any other Black background | 5 | 0.01% | 0.01% |
| Asian or Asian British | 1 | 0.00% | 0.00% |
| Black or Black British | 0 | 0.00% | 0.00% |
| BMI | 26.53 (SD 4.4) |  |  |
| Smoking Status |  |  |  |
| Current | 1662 | 3.3% | 3.3% |
| Previous | 17005 | 33.8% | 34.0% |
| Never | 31132 | 61.9% | 62.3% |
| Prefer not to answer | 186 | 0.4% | 0.4% |
| Alcohol consumption |  |  |  |
| Daily or almost daily | 8545 | 17.0% | 17.1% |
| Three or four times per week | 13961 | 27.7% | 27.9% |
| Once or twice a week | 13162 | 26.2% | 26.3% |
| One to three times per month | 5677 | 11.3% | 11.4% |
| Special Occasions | 5196 | 10.3% | 10.4% |
| Never | 3425 | 6.8% | 6.9% |
| Prefer not to answer | 19 | <0.1% | <0.1% |
| Leg Pain when standing still or sitting |  |  |  |
| Yes | 5246 | 10.4% | 52.5% |
| No | 4398 | 8.7% | 44.3% |
| Do not know | 315 | 0.6% | 3.1% |
| Prefer not to answer | 4 | <0.1% | <0.1% |
| Leg Pain when Walking |  |  |  |
| Yes | 9963 | 19.8% | 19.9% |
| No | 39643 | 78.8% | 79.3% |
| Do not know | 304 | 0.6% | 0.6% |
| Prefer not to answer | 75 | 0.1% | 0.2% |
| Leg Pain when Walking uphill or hurrying |  |  |  |
| Yes | 5856 | 11.6% | 58.8% |
| No | 3703 | 7.4% | 37.2% |
| Do not know | 387 | 0.8% | 3.9% |
| Prefer not to answer | 17 | <0.1% | 0.2% |
| Total thigh fat-free muscle volume | 10.15 (SD 2.52) |  |  |
| Total adipose tissue volume | 20.86 (SD 7.03) |  |  |
| Total lean tissue volume | 24.10 (SD 4.79) |  |  |
| Muscle fat infiltration  (mean of anterior left and right only) | 7.32 (SD 1.87) |  |  |
| Weight-to-muscle ratio | 7.62 (SD 1.33) |  |  |
| Total BMD | 1.20 (SD 0.15) |  |  |
| Total BMD (T-Score) | 0.59 (SD 1.24) |  |  |
| Femur total BMD (left) | 0.99 (SD 0.16) |  |  |
| Femur total BMD (right) | 1.00 (SD 0.16) |  |  |
| Leg BMD (left) | 1.23 (SD 0.18) |  |  |
| Leg BMD (right) | 1.24 (SD 0.18) |  |  |
| Legs BMD | 1.23 (SD 0.18) |  |  |
| Pelvis BMD | 1.00 (SD 0.15) |  |  |
| Hand grip strength (left) | 28.25 (SD 10.54) |  |  |
| Hand grip strength (right) | 30.41 (SD 10.58) |  |  |
| Duration of heavy DIY |  |  |  |
| Less than 15 minutes | 1332 | 2.7% | 5.6% |
| Between 15 and 30 minutes | 4148 | 8.2% | 17.4% |
| Between 30 minutes and 1 hour | 6428 | 12.8% | 26.9% |
| Between 1 hour and 1.5 hours | 4252 | 8.5% | 17.8% |
| Between 1.5 hours and 2 hours | 2793 | 5.6% | 11.7% |
| Between 2 and 3 hours | 2208 | 4.4% | 9.2% |
| Over 3 hours | 1436 | 2.9% | 6.0% |
| Do not know | 1168 | 2.3% | 4.9% |
| Prefer not to answer | 139 | 0.3% | 0.6% |
| Duration of light DIY |  |  |  |
| Less than 15 minutes | 1340 | 2.7% | 4.5% |
| Between 15 and 30 minutes | 6662 | 13.2% | 22.3% |
| Between 30 minutes and 1 hour | 9698 | 19.3% | 32.5% |
| Between 1 hour and 1.5 hours | 5382 | 10.7% | 18.1% |
| Between 1.5 hours and 2 hours | 3003 | 6.0% | 10.1% |
| Between 2 and 3 hours | 1953 | 3.9% | 6.6% |
| Over 3 hours | 997 | 2.0% | 3.3% |
| Do not know | 734 | 1.5% | 2.5% |
| Prefer not to answer | 46 | 0.1% | 0.2% |
|  |  |  |  |
| Duration of moderate activity | 64.17 (SD 64.79) |  |  |
| Duration of other exercises |  |  |  |
| Less than 15 minutes | 799 | 1.6% | 2.9% |
| Between 15 and 30 minutes | 4018 | 8.0% | 14.7% |
| Between 30 minutes and 1 hour | 12027 | 23.9% | 44.1% |
| Between 1 hour and 1.5 hours | 6221 | 12.3% | 22.8% |
| Between 1.5 hours and 2 hours | 1937 | 3.9% | 7.1% |
| Between 2 and 3 hours | 1075 | 2.1% | 3.9% |
| Over 3 hours | 930 | 1.9% | 3.4% |
| Do not know | 220 | 0.4% | 0.8% |
| Prefer not to answer | 43 | 0.1% | 0.2% |
| Duration of vigorous activity | 43.05 (SD 40.68) |  |  |
| Duration of walks | 59.61 (SD 63.95) |  |  |
| Duration of walks for pleasure |  |  |  |
| Less than 15 minutes | 299 | 0.6% | 0.7% |
| Between 15 and 30 minutes | 5856 | 11.6% | 14.3% |
| Between 30 minutes and 1 hour | 14906 | 29.6% | 36.3% |
| Between 1 hour and 1.5 hours | 8952 | 17.8% | 21.8% |
| Between 1.5 hours and 2 hours | 4717 | 9.4% | 11.5% |
| Between 2 and 3 hours | 3197 | 6.4% | 7.8% |
| Over 3 hours | 3003 | 6.0% | 7.3% |
| Do not know | 84 | 0.2% | 0.2% |
| Prefer not to answer | 8 | <0.1% | <0.1% |
| Frequency of heavy DIY in the last 4 weeks |  |  |  |
| Once in the last 4 weeks | 4023 |  | 18.7% |
| 2-3 times in the last 4 weeks | 6446 |  | 29.9% |
| Once a week | 5341 |  | 24.8% |
| 2-3 times per week | 3520 |  | 16.3% |
| 4-5 times per week | 786 |  | 3.6% |
| Every day | 207 |  | 1.0% |
| Do not know | 1123 |  | 5.2% |
| Prefer not to answer | 113 |  | 0.5% |
| Frequency of light DIY in the last 4 weeks |  |  |  |
| Once in the last 4 weeks | 2023 | 4.0% | 6.8% |
| 2-3 times in the last 4 weeks | 7097 | 14.1% | 23.8% |
| Once a week | 6004 | 11.9% | 20.2% |
| 2-3 times per week | 8873 | 17.6% | 29.8% |
| 4-5 times per week | 3214 | 6.4% | 10.8% |
| Every day | 1909 | 3.8% | 6.4% |
| Do not know | 653 | 1.3% | 2.2% |
| Prefer not to answer | 42 | 0.1% | 0.1% |
| Frequency of other exercises in last 4 weeks |  |  |  |
| Once in the last 4 weeks | 1395 | 2.8% | 5.1% |
| 2-3 times in the last 4 weeks | 3911 | 7.8% | 14.3% |
| Once a week | 5178 | 10.3% | 19.0% |
| 2-3 times per week | 10891 | 21.6% | 39.9% |
| 4-5 times per week | 4545 | 9.0% | 16.7% |
| Every day | 1035 | 2.1% | 3.8% |
| Do not know | 262 | 0.5% | 1.0% |
| Prefer not to answer | 53 | 0.1% | 0.2% |
| Frequency of stair climbing in the last 4 weeks | |  |  |
| None | 4369 | 8.7% | 8.8% |
| 1-5 times a day | 8202 | 16.3% | 16.4% |
| 6-10 times a day | 17802 | 35.4% | 35.7% |
| 11-15 times a day | 10483 | 20.8% | 21.0% |
| 16-20 times a day | 5187 | 10.3% | 10.4% |
| More than 20 times a day | 3734 | 7.4% | 7.5% |
| Do not know | 148 | 0.3% | 0.3% |
| Prefer not to answer | 17 | <0.1% | <0.1% |
| Frequency of strenuous sports in the last 4 weeks | |  |  |
| Once in the last 4 weeks | 219 | 0.4% | 3.6% |
| 2-3 times in the last 4 weeks | 752 | 1.5% | 12.3% |
| Once a week | 1342 | 2.7% | 22.0% |
| 2-3 times per week | 2690 | 5.3% | 44.0% |
| 4-5 times per week | 939 | 1.9% | 15.4% |
| Every day | 104 | 0.2% | 1.7% |
| Do not know | 58 | 0.1% | 1.0% |
| Prefer not to answer | 8 | <0.1% | 0.1% |
| Frequency of walks for pleasure in the last 4 weeks | |  |  |
| Once in the last 4 weeks | 1708 | 3.4% | 4.2% |
| 2-3 times in the last 4 weeks | 9022 | 17.9% | 22.0% |
| Once a week | 6682 | 13.3% | 16.3% |
| 2-3 times per week | 11191 | 22.2% | 27.3% |
| 4-5 times per week | 6317 | 12.6% | 15.4% |
| Every day | 6027 | 12.0% | 14.7% |
| Do not know | 70 | 0.1% | 0.2% |
| Prefer not to answer | 5 | <0.1% | <0.1% |
| Number of days per week of moderate physical activity (10+ mins) | 4.06 (SD 2.21) |  |  |
| Number of days per week of vigorous physical activity (10+ mins) | 2.01 (SD 1.91) |  |  |
| Number of days per week walked (10+ mins) | 5.75 (SD 1.70) |  |  |
| Types of physical activity in last 4 weeks |  |  |  |
| Walking for pleasure (not as a means of transport) | 41022 | 81.5% | 31.7% |
| Other exercises (e.g., swimming, cycling, keep fit, bowling) | 27270 | 54.2% | 21.1% |
| Strenuous sports | 6112 | 12.1% | 4.7% |
| Light DIY (e.g., pruning, watering the lawn) | 29815 | 59.2% | 23.1% |
| Heavy DIY (e.g., weeding, lawn mowing, carpentry, digging) | 23904 | 47.5% | 18.5% |
| None of the above | 1215 | 2.4% | 0.9% |
| Prefer not to answer | 19 | <0.1% | <0.1% |
| Usual walking pace |  |  |  |
| Slow Pace | 2513 | 5.0% | 5.03% |
| Steady Average pace | 25257 | 50.2% | 50.57% |
| Brisk Pace | 22135 | 44.0% | 44.32% |
| None of the above | 31 | <0.1% | <0.1% |
| Prefer not to answer | 6 | <0.1% | <0.1% |
| Alcohol Units (per day) | 1.08 (SD 1.21) |  |  |
| BMD = Bone Mineral Density, BMI = Body Mass Index, SD = Standard Deviation | | | |

**Table 3A.** Association between fat-free muscle volume and outcomes unadjusted (Model 1) and adjusted for age and sex (Model 2)

| **Outcomes** | **Model 1** | **Model 2** |
| --- | --- | --- |
| Bone mineral density | 0.041 (0.041 to 0.041) | 0.032 (0.031 to 0.033) |
| Hand grip strength |  |  |
| Left | 3.02 (2.99 to 3.05) | 1.58 (1.53 to 1.63) |
| Right | 3.01 (2.98 to 3.04) | 1.56 (1.51 to 1.61) |
| Smoking status, OR (95% CI) | 1.06 (1.04 to 1.09) | 0.92 (0.88 to 0.96) |
| Alcohol use, OR (95% CI) | 1.13 (1.12 to 1.14) | 1.06 (1.04 to 1.08) |
| Leg pain |  |  |
| Leg pain on walking, OR (95% CI) | 1.00 (0.99 to 1.01) | 1.05 (1.03 to 1.07) |
| Leg pain on walking: action taken, OR (95% CI) | 0.99 (0.96 to 1.03) | 0.92 (0.87 to 0.98) |
| Leg pain on walking: effect of standing still, OR (95% CI) | 0.98 (0.95 to 1.00) | 1.00 (0.96 to 1.04) |
| Leg pain when walking ever disappears while walking, OR (95% CI) | 1.02 (0.99 to 1.05) | 1.03 (0.99 to 1.09) |
| Leg pain when walking normally, OR (95% CI) | 0.98 (0.96 to 1.00) | 0.98 (0.95 to 1.02) |
| Leg pain when walking uphill or hurrying, OR (95% CI) | 0.93 (0.91 to 0.95) | 0.92 (0.88 to 0.95) |
| Leg pain in calf/calves, OR (95% CI) | 1.00 (0.98 to 1.02) | 0.92 (0.88 to 0.96) |
| Leg pain when standing still or sitting, OR (95% CI) | 0.98 (0.96 to 1.00) | 1.00 (0.96 to 1.03) |
| Physical activity |  |  |
| Duration of walks | -0.53 (-0.81 to -0.24) | -0.88 (-1.40 to -0.35) |
| Duration walking for pleasure, OR (95% CI) | 1.01 (1.00 to 1.02) | 1.05 (1.04 to 1.07) |
| Duration of moderate activity | -0.52 (-0.81 to -0.22) | 0.13 (-0.41 to 0.67) |
| Duration of vigorous activity | 1.04 (0.84 to 1.25) | 1.40 (1.02 to 1.78) |
| Duration of other exercises, OR (95% CI) | 1.04 (1.03 to 1.05) | 1.09 (1.07 to 1.12) |
| Duration of strenuous sports, OR (95% CI) | 1.04 (1.02 to 1.06) | 1.01 (0.97 to 1.05) |
| Duration of light DIY, OR (95% CI) | 1.05 (1.04 to 1.07) | 1.02 (1.00 to 1.04) |
| Duration of heavy DIY, OR (95% CI) | 1.08 (1.06 to 1.09) | 1.05 (1.03 to 1.07) |
| Frequency of light DIY in last 4 weeks, OR (95% CI) | 0.99 (0.98 to 1.00) | 1.00 (0.98 to 1.02) |
| Frequency of heavy DIY in last 4 weeks, OR (95% CI) | 1.03 (1.02 to 1.04) | 1.01 (0.99 to 1.03) |
| Frequency of other exercises in last 4 weeks, OR (95% CI) | 1.06 (1.05 to 1.07) | 1.10 (1.08 to 1.12) |
| Frequency of stair climbing in last 4 weeks, OR (95% CI) | 1.00 (0.99 to 1.01) | 1.01 (1.00 to 1.02) |
| Frequency of strenuous sports in last 4 weeks, OR (95% CI) | 1.04 (1.02 to 1.07) | 1.11 (1.07 to 1.15) |
| Frequency of walking for pleasure in last 4 weeks, OR (95% CI) | 0.98 (0.97 to 0.99) | 0.97 (0.96 to 0.99) |
| BMD = Bone Mineral Density, BMI = Body Mass Index, DIY = Do it yourself, OR = Odds Ratio, SD = Standard Deviation | | |

**Table 4A.** Association between Intramuscular Fat and Outcomes unadjusted (Model 1) and adjusted for age and sex (Model 2)

| **Outcomes** | **Model 1** | **Model 2** |
| --- | --- | --- |
| Bone mineral density | -0.013 (-0.014 to -0.012) | 0.0064 (0.0056 to 0.0072) |
| Hand grip strength |  |  |
| Left | -1.86 (-1.91 to -1.80) | -0.50 (-0.54 to -0.45) |
| Right | -1.87 (-1.92 to -1.81) | -0.51 (-0.55 to -0.46) |
| Smoking status, OR (95% CI) | 1.05 (1.02 to 1.08) | 1.16 (1.13 to 1.20) |
| Alcohol use, OR (95% CI) | 0.93 (0.92 to 0.94) | 0.96 (0.95 to 0.97) |
| Leg pain |  |  |
| Leg pain on walking, OR (95% CI) | 1.26 (1.24 to 1.28) | 1.28 (1.26 to 1.30) |
| Leg pain on walking: action taken, OR (95% CI) | 1.21 (1.17 to 1.25) | 1.22 (1.18 to 1.27) |
| Leg pain on walking: effect of standing still, OR (95% CI) | 1.05 (1.02 to 1.08) | 1.07 (1.04 to 1.10) |
| Leg pain when walking ever disappears while walking, OR (95% CI) | 0.87 (0.84 to 0.90) | 0.85 (0.82 to 0.88) |
| Leg pain when walking normally, OR (95% CI) | 1.10 (1.07 to 1.12) | 1.11 (1.08 to 1.14) |
| Leg pain when walking uphill or hurrying, OR (95% CI) | 1.14 (1.11 to 1.17) | 1.15 (1.12 to 1.18) |
| Leg pain in calf/calves, OR (95% CI) | 1.04 (1.02 to 1.07) | 1.07 (1.04 to 1.10) |
| Leg pain when standing still or sitting, OR (95% CI) | 1.05 (1.02 to 1.07) | 1.06 (1.03 to 1.10) |
| Physical activity |  |  |
| Duration of walks | -0.98 (-1.37 to -0.58) | -0.95 (-1.38 to -0.52) |
| Duration walking for pleasure, OR (95% CI) | 0.91 (0.89 to 0.92) | 0.87 (0.86 to 0.88) |
| Duration of moderate activity | 0.057 (-0.35 to 0.47) | -1.19 (-1.63 to -0.74) |
| Duration of vigorous activity | -1.75 (-2.05 to -1.45) | -1.53 (-1.86 to -1.20) |
| Duration of other exercises, OR (95% CI) | 0.94 (0.92 to 0.95) | 0.92 (0.91 to 0.94) |
| Duration of strenuous sports, OR (95% CI) | 1.05 (1.01 to 1.09) | 1.03 (0.99 to 1.07) |
| Duration of light DIY, OR (95% CI) | 0.96 (0.94 to 0.97) | 0.98 (0.97 to 0.99) |
| Duration of heavy DIY, OR (95% CI) | 0.92 (0.91 to 0.94) | 0.96 (0.94 to 0.98) |
| Frequency of light DIY in last 4 weeks, OR (95% CI) | 1.01 (1.00 to 1.03) | 0.98 (0.96 to 0.99) |
| Frequency of heavy DIY in last 4 weeks, OR (95% CI) | 0.98 (0.96 to 0.99) | 0.96 (0.94 to 0.98) |
| Frequency of other exercises in last 4 weeks, OR (95% CI) | 0.88 (0.86 to 0.89) | 0.87 (0.86 to 0.88) |
| Frequency of stair climbing in last 4 weeks, OR (95% CI) | 0.87 (0.87 to 0.88) | 0.86 (0.85 to 0.87) |
| Frequency of strenuous sports in last 4 weeks, OR (95% CI) | 0.86 (0.83 to 0.89) | 0.85 (0.82 to 0.89) |
| Frequency of walking for pleasure in last 4 weeks, OR (95% CI) | 1.00 (0.99 to 1.02) | 0.99 (0.97 to 1.00) |
| BMD = Bone Mineral Density, BMI = Body Mass Index, OR = Odds Ratio, SD = Standard Deviation | | |
